# Contraction Dynamics of Rod Microtissues of Gingiva-Derived and Periodontal Ligament-Derived Cells

**DOI:** 10.3389/fphys.2018.01683

**Published:** 2018-12-21

**Authors:** Gunpreet Oberoi, Klara Janjić, Anna Sonja Müller, Barbara Schädl, Oleh Andrukhov, Andreas Moritz, Hermann Agis

**Affiliations:** ^1^Department of Conservative Dentistry and Periodontology, University Clinic of Dentistry, Medical University of Vienna, Vienna, Austria; ^2^Austrian Cluster for Tissue Regeneration, Vienna, Austria; ^3^Center for Medical Physics and Biomedical Engineering, Medical University Vienna, Vienna, Austria; ^4^Ludwig Boltzmann Institute for Experimental and Clinical Traumatology, Vienna, Austria

**Keywords:** contraction dynamics, microtissue, rods, cell-signaling pathways, scaffold-free, periodontal regeneration, 3D culture

## Abstract

Tissue engineering strategies using microtissues as “building blocks” have high potential in regenerative medicine. Cognition of contraction dynamics involved in the *in vitro* self-assembly of these microtissues can be conceived as the bedrock of an effective periodontal tissue regenerative therapy. Our study was directed at evaluating the shrinkage in the rod-shaped structure of a directed self-assembly of human gingiva-derived cells (GC) and periodontal ligament-derived cells (PDLC) and developing insights into the potential mechanisms responsible for the shrinkage. GC and PDLC were seeded in non-adherent agarose molds to form rod microtissues. Cells used for the experiments were characterized using fluorescence-activated cell sorting (FACS). To assess the viability, resazurin-based cytotoxicity assays, trypan blue dye exclusion assay, MTT and live/dead staining, and histological evaluation of rods based on hematoxylin and eosin staining were performed. Rod contraction was evaluated and measured at 0, 2, 6, and 24 h and compared to L-929 cells. The role of transforming growth factor (TGF)-β signaling, phosphoinositide 3-kinase (PI3K)/AKT, and mitogen activated protein kinase (MAPK) signaling was analyzed. Our results show that the rod microtissues were vital after 24 h. A reduction in the length of rods was seen in the 24 h period. While the recombinant TGF-β slightly reduced contraction, inhibition of TGF-β signaling did not interfere with the contraction of the rods. Interestingly, inhibition of phosphoinositide 3-kinase by LY294002 significantly delayed contraction in GC and PDLC rods. Overall, GC and PDLC have the ability to form rod microtissues which contract over time. Thus, approaches for application of these structures as “building blocks” for periodontal tissue regeneration should consider that rods have the capacity to contract substantially. Further investigation will be needed to unravel the mechanisms behind the dynamics of contraction.

## Introduction

Cells have the potential to self-assemble into spheroid-shaped structures *in vitro* (Dean et al., 2007). This self-aggregation is effected on the one hand by surface membrane proteins and signaling molecules regulating intercellular cohesiveness (Dean and Morgan, 2008; Barone and Heisenberg, 2012) and on the other hand by the mechanical properties of microenvironment and extracellular matrix (Kaufman et al., 2016; Kaufman and Skrtic, 2017). The ability of cells to form spheroides has been employed to fabricate microtissues as “building blocks” for tissue engineering in bone defects (Langenbach et al., 2010, 2013; Ivanov and Grabowska, 2018). Due to the *in vivo* mimicking properties these microtissues are also used for screening and characterization approaches (Langenbach et al., 2010, 2013; Ivanov and Grabowska, 2018). From existing literature, it is evident that different cell types have wide-ranging magnitude and rate of self-aggregation (Svoronos et al., 2014). Individual cells migrate to form loose aggregates of three dimensional tissue structures with specific x, y, and z dimensions (Svoronos et al., 2014). Miscellaneous human and murine cells have previously been directed to assemble into complex 3D structures like spheroids, rods, tori, and honeycombs with varying stability and morphological alterations over time (Dean et al., 2007). *In vivo* a capillary network is required to diffuse oxygen and nutrients to thicker tissues (Griffith et al., 2005). The basic microtissues lack this network. However, unlike a spheroid of which the final dimensions are governed by diffusion limit, rods can be designed in any length as long as their width permits capillary diffusion (Dean et al., 2007). To establish successful functionality and biosimulation of the replaced engineered tissue, it is imperative to understand its properties (Blumlein et al., 2017).

The population dynamics of scaffolds from 3D gingival fibroblast-derived structures has been thoroughly studied in the past (Agis et al., 2014), while similar studies for scaffold-free approaches involving cells derived from periodontal tissue is yet to be unraveled. In our study, we directed self-aggregation of human gingiva-derived cells (GC), periodontal ligament-derived cells (PDLC), and L-929 cells into rod-shaped structures with the aid of non-adherent agarose molds that do not encroach the intercellular cohesive mechanisms (Svoronos et al., 2014) to quantify the contraction kinetics and geometry of rod microtissues.

Transforming growth factor beta (TGF-β), belonging to the cytokine family is involved in the regulation of cellular functions such as cell differentiation, migration, adhesion, survival, and conditioning of developmental outcome in healthy and diseased tissue (Inman et al., 2002). Both serine/threonine kinases constitute Type I and II receptor complexes involved in TGF-β signaling pathways. SB-431542 has been identified as an inhibitor of activin receptor-like TGF-β Type I receptor specifically activin receptor-like kinase (ALK5) (Halder et al., 2005). TGF-β is secreted by periodontal ligament-derived mesenchymal stem cells (Li et al., 2014) and is also produced in periodontal ligament fibroblasts under stress to induce cellular responses by autocrine mechanisms. TGF-β has been revealed to induce the differentiation of gingival fibroblasts into myofibroblasts which in turn generate contractile forces for wound closure (Vaughan et al., 2000; Komatsu et al., 2016) and activate mitogen activated protein kinase (MAPK) signaling pathways (Halder et al., 2005).

Mitogen activated protein kinase including extracellular signal-regulated kinase (ERK), p38, and c-Jun N-terminal kinase (JNK), and the phosphoinositide 3-kinase (PI3K) are protein kinases involved in intra-cellular signaling pathways responsible for cellular proliferation, differentiation, mitogenic activity, and regeneration of fibroblastic cells (Gruber et al., 2004; Agis et al., 2010). To gain insights into the involved signaling mechanisms we applied MAPK and PI3K inhibitors, and TGF-β to investigate their effect in modulating the microtissue rod geometry.

Our main objective was to measure the dimensional shrinkage in the rod geometry over time and apply this information to help to bridge the gap from *in vitro* scaffold-free functional microtissue production to their utilization in periodontal defects *in vivo*.

## Materials and Methods

### Cell Culture and Isolation

A previously established protocol was used to isolate GC and PDLC (Agis et al., 2012). When informed and written consent were given, the extracted 3rd molars, without a prior history of pulpitis or periodontitis were employed to prepare GC and PDLC. The soft tissues accompanying the dental cervix and root were processed to harvest GC and PDLC, respectively. α-minimal essential medium (Gibco, Invitrogen Corporation, Carlsbad, CA, United States), supplemented with 10% fetal bovine serum (FBS, LifeTech, Vienna, Austria) and antibiotics (Gibco, Invitrogen Corporation, United States) at 37°C, 5% CO_2_, and 95% humidity was used for expanding and cultivating GC and PDLC. GC and PDLC were not used beyond passage 7 (Janjić et al., 2017b). The protocol was approved by the ethics committee of the Medical University of Vienna (631/2007). L-929 cells (ATCC, Rockville, MD, United States) were cultured under the same conditions.

### Flow Cytometry

Gingiva-derived cells and PDLC from three donors each were characterized with fluorescent activated flow cytometry using BD fluorescence-activated cell sorting (FACS) Calibur^TM^ equipment using a pre-established protocol (Andrukhov et al., 2016). GC and PDLC at passage 3 were trypsinized and at least 5 × 10^4^ cells/mL were transferred to flow cytometry test tubes. Cells were washed with flow cytometry buffer consisting of PBS supplemented with 3% BSA and 0.09% NaN_3_, and incubated with monoclonal antibodies for mesenchymal stem cell surface markers with: phycoerythrin (PE)-conjugated mouse anti-human CD29, PE-conjugated mouse anti-human CD73, PE-conjugated mouse anti-human CD90, PE-conjugated mouse anti-human CD105, PE-conjugated mouse anti-human CD146, and for hematopoietic stem cell (HSC) surface markers with FITC-conjugated mouse anti-human CD34, and FITC-conjugated mouse anti-human CD45. FITC-conjugated mouse IgG1 K and PE-conjugated mouse IgG1 K were used as isotype controls. Acquisition was limited to 3.000 events.

### Rods

3D rod cultures were created using 3D Petri dishes^®^ (Microtissues, Inc., Providence, RI, United States) following the protocol of the manufacturer. The agarose molds were conditioned with cell culture medium and placed into 24 well plates where cell suspensions of 480,000 cells in drops of 70 μL were pipetted into the molds. Each well then received 1 mL of cell culture medium and plates were incubated for 24 h.

### TGF-β Signaling

To investigate the role of TGF-β on the contraction of the microtissues formed by GC and PDLC, the cells were incubated with human 10 ng/mL TGF-β or its inhibitor SB431542 at 10 μM. The cells were incubated as described above. Images were taken after 24 h using a light microscope with the 4-fold objective and the OptoCapture software 2.2 (OPTOTEAM, Vienna, Austria). Length and width were calculated in millimeters using Call EZ software for MacOS. Data were presented as compared to the untreated cells, referred to as control.

### MAPK and PI3/AKT Signaling

To investigate the role of MAPK and PI3K signaling, GC and PDLC were incubated with the pharmacological inhibitors LY294002 (PI3K-inhibitor; Sigma), SB203580 (p38-inhibitor; Sigma), SP600125 (JNK-inhibitor; Calbiochem, San Diego, CA, United States), and U0126 (ERK-inhibitor; Cell Signaling Technology, Beverly, MA, United States), all at 10 μM for 24 h as described above. Images were taken under a light microscope using the 4-fold objective and OptoCapture software 2.2 after 24 h of incubation. Length and width were calculated in millimeters using Call EZ software. Data were presented as compared to the untreated cells, referred to as control.

### Trypan Blue Dye Exclusion Assay

To count the number of live and dead cells in the rods after the 24 h incubation period, we used the trypan blue dye exclusion assay. Medium was sucked out carefully leaving the rods intact in the molds. The rods were washed with PBS and transferred into tubes. The tubes were then centrifuged. After removing of the PBS the rods in the pellet were trypzinised. Trypsin activity was stopped with serum containing medium and centrifuged. Then the pellet was resuspended in medium and stained in Trypan blue in 1:1 concentration. Vital cells remained unstained while dead cells appeared blue stained. Vital cells and dead cells were counted manually.

### MTT Staining

Gingiva-derived cells, PDLC, and L-929 rods were incubated with 1 mg/mL MTT at 37°C for 30 min in a 24-well plate, post 24 h incubation period. Formazan formation was observed under a light microscope and images were taken (Kurzmann et al., 2017).

### Live/Dead Staining

To assess the viability of GC, PDLC, and L-929 rods, samples were stained with the Live/Dead Cell Staining Kit (Enzo Life Sciences AG, Lausen, TX, United States) following the manufacturer’s guidelines in a 24 well plate after the 24 h incubation period (Janjić et al., 2018a). The vitality of the rods was analyzed using fluorescence microscopy for green and red, with a B-2A filter (excitation filter wavelengths: 450–490 nm), respectively. Vital cells became visible as green while the dead cells were red.

### Resazurin-Based Toxicity Assay

To measure the viability of the GC and PDLC rod microtissues, a resazurin-based toxicity assay was done according to the protocol given by the manufacturer. Resazurin dye solution (Merck, Darmstadt, Germany) was added into each well of the 24 well plate, 24 h after seeding the cells into agarose molds at a final concentration of 10%. Post-incubation at 37°C for 8 h, fluorescence was evaluated using a Synergy HTX multi-mode reader (BioTek, Winooski, VT, United States) at a wavelength of 600 nm, using an excitation wavelength of 540 nm (Müller et al., 2010). The data were obtained relative to the control.

### Time-Bound Contraction

To assess the dynamics of rod formation over time, we measured morphological and geometrical changes in length and width of the rod microtissues. Images of the self-assembly of GC and PDLC in the agarose molds were taken at 0 (immediately after seeding), 2, 6, and 24 h after seeding as described before. Length and width was calculated in millimeters using the Call EZ software for MacOS.

### Histological Evaluation

Hematoxylin-eosin (HE) staining of GC and PDLC rods was performed after a 24 h incubation period using the following protocol. Samples were fixed in 4% acid-free neutral buffered formalin, washed, and dehydrated. Then the samples were embedded in paraffin and cut using a rotary microtome. The slices were stained 7 min in Mayer’s hematoxylin and washed in aqua dest. After differentiating with 0.1% HClOH the slides were washed for 10 min in tap water. Next, they were stained in 0.5% Eosin G (with two droplets of glacial acetic acid), and rinsed in aqua dest, followed by dehydration in 70% EtOH, 96% EtOH, 100% EtOH, and xylene. Finally, they were permanently embedded.

### Statistical Evaluation

Statistical analysis was done with IBM SPSS Statistics Version 24 (IBM Corporation, Armonk, NY, United States), using the ANOVA and *post hoc* Dunnet test. The level of significance was set at *p* < 0.05.

## Results

### Characterization of Gingiva-Derived Cells and Periodontal-Ligament-Derived Cells

The percentage of positively stained cells for surface markers of mesenchymal stem cells and hematopoietic stem cells on GC and PDLC is represented in Table 1. As it can be seen, cells were positively stained for mesenchymal markers CD29, CD73, CD90, and CD105, and negative for hematopoietic markers, CD34 and CD45. The expression of the mesenchymal marker CD146 exhibited large variability between donors of GC and PDLC ranging from 1.92 ± 0.22% to 3.31 ± 0.47% and 1.36 ± 0.46% to 20.60 ± 2.88%, respectively.

**Table 1:** Cell surface characterization of gingiva-derived cells (GC) and periodontal ligament-derived cells (PDLC) [mean ± standard deviation, in percent (%)].

	MSC					HSC	
	CD29	CD73	CD90	CD105	CD146	CD34	CD45
GC Donor 1	98.89 ± 0.00	99.16 ± 0.00	98.92 ± 0.52	97.78 ± 0.41	3.31 ± 0.47	2.92 ± 0.74	3.39 ± 0.20
GC Donor 2	99.19 ± 0.03	98.92 ± 0.25	99.09 ± 0.18	97.11 ± 0.35	2.68 ± 0.36	5.10 ± 0.28	4.20 ± 0.28
GC Donor 3	99.26 ± 0.01	99.29 ± 0.24	99.49 ± 0.20	98.02 ± 0.02	1.92 ± 0.22	3.08 ± 0.01	2.21 ± 0.15
PDLC Donor 1	99.32 ± 0.05	98.94 ± 0.23	99.21 ± 0.20	98.10 ± 0.01	20.60 ± 2.88	1.97 ± 0.17	1.79 ± 0.23
PDLC Donor 2	98.29 ± 0.45	98.27 ± 0.13	98.20 ± 0.19	96.85 ± 0.72	3.27 ± 0.00	2.43 ± 0.05	2.35 ± 0.14
PDLC Donor 3	98.40 ± 0.01	98.89 ± 0.25	99.08 ± 0.39	98.22 ± 0.05	1.36 ± 0.46	3.08 ± 0.01	2.50 ± 0.13


*Flow cytometry was performed for the mesenchymal stem cell (MSC) surface markers and hematopoietic stem cell (HSC) surface markers on GC and PDLC. Each sample was measured twice. The mean ± standard deviation of the gated percentage of positively stained cells is displayed in the table.*

### Contraction Dynamics of Rods From Gingiva-Derived Cells, Periodontal-Ligament-Derived Cells, and L-929

**FIGURE 1:**
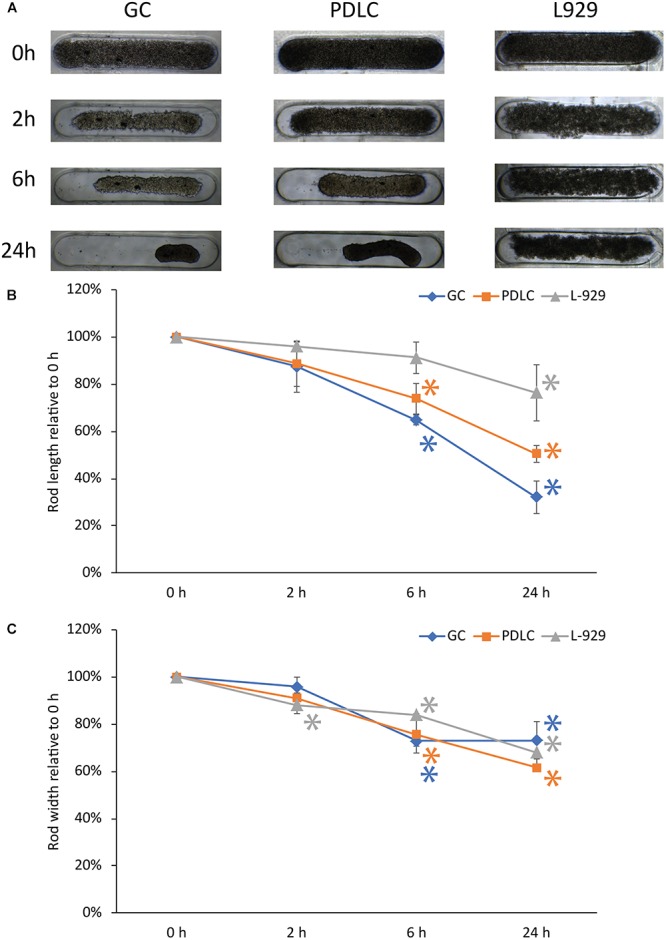
Contraction dynamics of rods of gingiva and periodontal ligament-derived cells, and L-929. 3D rod microtissue cultures were created using 3D Petri dishes^®^ with gingiva-derived cells (GC) periodontal ligament-derived cells (PDLC), and L-929 cells. Images were taken at directly after seeding (0 h), at 2, 6, and 24 h after seeding **(A)**. The length **(B)**, and width **(C)** was quantified. Experiments were performed three times and four rods per experiment were analyzed. The data points show the length and with relative to 0 h as mean ± standard deviation. ^∗^*p* < 0.05.

Rods from GC and PDLC contracted over time, while L-929 behaved differently in terms of microtissue formation and contraction (Figure 1A). To quantify the kinetics of rod formation over a 24 h time period, we measured changes in the length (Figure 1B) and width (Figure 1C) of the rod microtissues. The analysis showed that the GC and PDLC rod length changed to approximately 32 and 50% of their original width after 24 h, respectively, (*p* < 0.05). The maximum contraction in the width was seen 24 h after incubation which was around 73 and 62% of the original length for GC and PDLC, respectively. L-929 did not show similar contraction behavior with regard to length which reached 76% after 24 h and still no stable rod microtissues had formed. With regard to width contraction, rods from all cell types followed similar dynamics.

### Rods Maintain Viability Based on Trypan Blue Dye Exclusion Assay, Live/Dead Staining, MTT Staining, Histological Analysis, and Resazurin-Based Toxicity Assay

**FIGURE 2:**
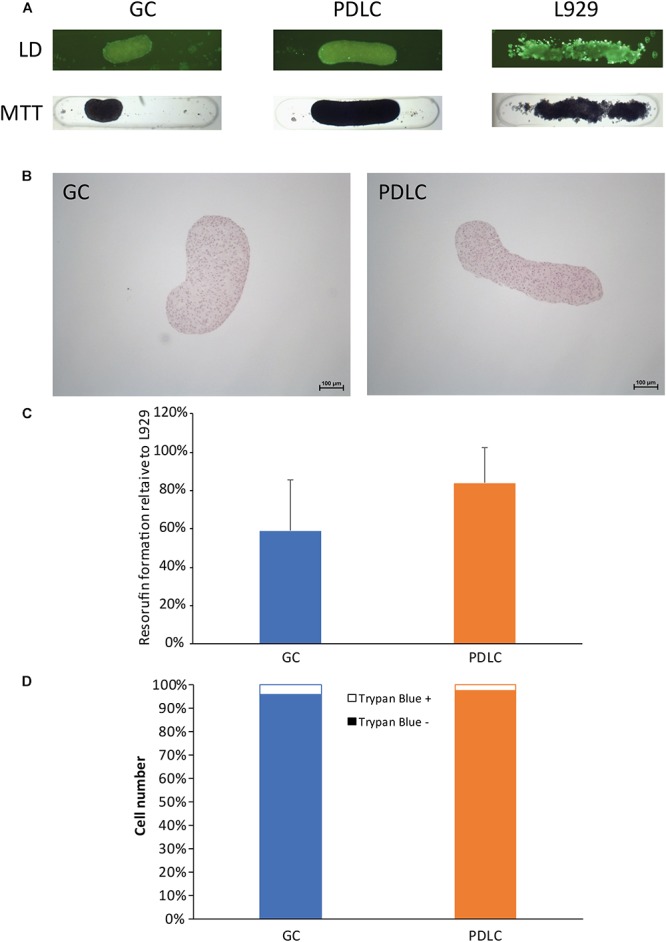
Rods maintain viability based on live/dead (LD) staining, MTT staining, histological analysis, and resazurin-based toxicity assay, and cell count. To assess the viability of the rod microtissues live/dead staining, the MTT staining **(A)**, and histological evaluation by hematoxylin- eosin staining **(B)** were performed with rods after 24 h. Furthermore resazurin-based toxicity assays **(C)**, and trypan exclusion assay **(D)** were done. Experiments were performed three times with four replicates each. **(C)** Bars represent mean ± standard deviation relative to L-929 cells. Gingiva-derived cells (GC); periodontal ligament-derived cells (PDLC).

To assess the viability of the microtissue rods the live/dead and the MTT staining procedures were performed (Figure 2). In the live/dead staining, the self-assembly of GC, PDLC, and L929 after labeling with fluorescent dye appeared as bright green rods after 24 h of incubation period, signifying the viability of the cell assembly (Figure 2A). The images of the MTT staining also showed that GC, PDLC, and L-929 rods turned dark blue indicating the conversion of the tetrazolium dye into formazan (Figure 2A).

To explore the histology of the microtissue rods, they were stained with hematoxylin and eosin dyes (Figure 2B). The GC rods appeared kidney bean-shaped structures with blue-violet nuclei and red cytoplasm while the PDLC rods appeared elongated cylindrical in shape with similarly stained cells.

To confirm the viability results from live/dead and MTT staining, the resazurin-based cytotoxicity assay was performed (Figure 2C). The fluorescence measurement data showed that the GC and PDLC rods were viable.

To further authenticate the above data, live/dead cell count was done from single cell suspension of rods, which confirmed that the contraction of rods was a result of cell compaction and not the decrease in viability. The data showed that the number of live cells in rods after 24 h incubation period ranged from 93.9 to 99.2% while the dead cells ranged from 0.8 to 6.0% (Figure 2D).

### TGF-β but Not the TGF-β Signaling Inhibitor SB43154 Modulates Contraction

**FIGURE 3:**
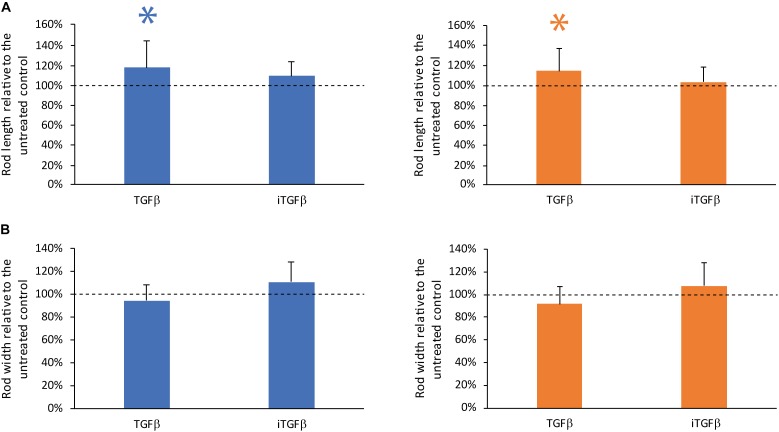
TGF-β but not the TGF-β signaling inhibitor SB43154 modulates contraction. To investigate the role of TGF-β on the contraction of the rod microtissues formed by gingiva-derived cells (GC) (blue bars) and periodontal ligament-derived cells (PDLC) (orange bars), the cells were incubated with human TGF-β or the TGF-β signaling inhibitor SB431542. After 24 h images were taken and length **(A)** and width **(B)** were calculated. Experiments were performed six times with four different donors in total. Bars represent mean ± standard deviation relative to untreated cells (Untreated control). ^∗^*p* < 0.05.

To investigate the role of TGF-β on the contraction dynamics of the rod microtissues were formed by GC and PDLC in the presence of recombinant human TGF-β and the TGF-β signaling inhibitor SB431542 (Figure 3). The addition of recombinant TGF-β showed a minor impact on the contraction of the GC and PDLC rods with regard to length but not width while the TGF-β signaling inhibitor without the addition of TGF-β did not have any significant effect on length and width of the rods after 24 h of incubation. These data suggest that TGF-β produced by the cells does not play a major role in the contraction but that externally provided active TGF-β might modulate the contraction process.

### Inhibitor of PI3K Pathway Affects Rod Geometry Significantly

**FIGURE 4:**
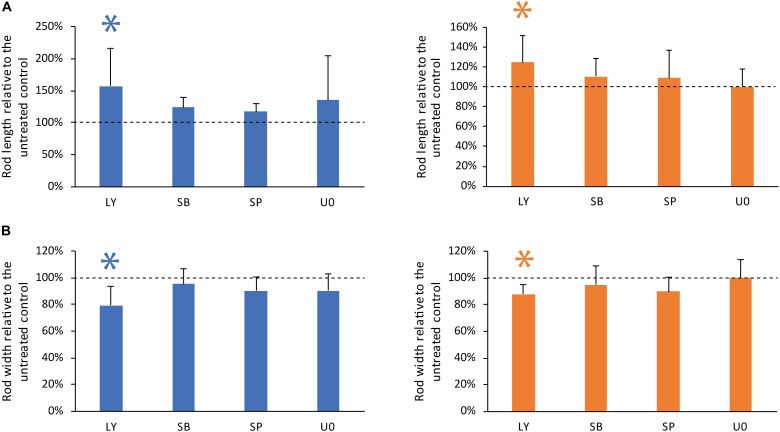
Inhibitor of PI3K pathway affects rod geometry significantly. To investigate the role of PI3K/AKT and mitogen activated protein kinase signaling, GC (blue bars) and PDLC (orange bars) were incubated with the pharmacological inhibitors LY294002 (LY, PI3K-inhibitor), SB203580 (SB, p38-inhibitor), SP600125 (SP, JNK-inhibitor), and U0126 (U0, ERK-inhibitor). Experiments were performed six times with four different donors in total. After 24 h images were taken and length **(A)** and width **(B)** was calculated. Bars represent mean ± standard deviation relative to untreated cells (Untreated control). ^∗^*p* < 0.05.

To investigate the tailoring of the self-assembly of GC and PDLC rods by MAPK and PI3K intracellular signaling pathways, GC and PDLC were incubated with the pharmacological inhibitors LY294002 (PI3K-inhibitor), SB203580 (p38-inhibitor), SP600125 (JNK-inhibitor), and U0126 (ERK-inhibitor), all at 10 μM for 24 h (Figure 4). The inhibitor of PI3K, LY294002, was witnessed to significantly increase the length and decrease the width of both gingival and periodontal rod microtissues (*p* < 0.05), suggesting the role of PI3K pathway in controlling the contraction dynamics of the microtissues *in vitro*.

## Discussion

In this descriptive study we investigated the contraction dynamics in the rod-shaped microtissues of cells derived from gingiva and periodontal ligament. We found that the cells used in the study fulfill the minimal surface maker criteria for mesenchymal stem cells (Dominici et al., 2006). Interestingly, CD146 was only detected in a low percentage of cells and exhibited large variability between donors. This result is in line with previous observation showing that periodontal ligament cells consist of several populations exhibiting either low, or high surface expression of CD146 (Huang et al., 2009; Xu et al., 2009). We found that these cells have the ability to form rod microtissues with rods compacting over time due to contraction of the assembly and not due to loss of viability, as confirmed by the trypan blue dye exclusion assay. Only 0.8–6.0% rod cells on an average displayed absorption of trypan blue due to membrane disruption (Schanne et al., 1979) and death while the majority remained unstained and vital. Addition of TGF-β but not TGF-β signaling inhibitor hindered the contraction, suggesting that active TGF-β can modulate the reduction in rod length but autocrine mechanisms are not the major player in this system. Interestingly inhibition of the PI3K/AKT pathway also interfered with the contraction of the rods in length and width suggesting that the pathway plays a key role in the contraction process.

Understanding the contraction dynamics involved in the *in vitro* self-assembly of oral microtissues can be conceived as the foundation of the application of microtissues for effective periodontal tissue regenerative approaches. Particularly in dentistry, spheroid microtissues are proposed as building blocks for dental pulp cell regeneration both *in vitro* and *ex vivo* (Janjić et al., 2017a, 2018b). Similar approaches were suggested for bone regeneration (Langenbach et al., 2010, 2013). The dynamics of spheroidal cell assembly of DU 145 human prostate cancer cells has been extensively studied using non-adherent agarose gel molds in liquid overlay cultures as they promote cell-to-cell cohesion, rather than cell-to-substrate adhesion (Enmon et al., 2001). Once, the energy minimization specifications are met, they mold themselves into alternate structures (Svoronos et al., 2014). By modeling the non-adhesive gel that favors self-assembly of cells (Youssef et al., 2011), energy differences in the resulting shapes can be altered and more intricate shapes like rods, toroids, and honeycombs can be obtained (Dean et al., 2007; Svoronos et al., 2014). Both, the cell-adhesion molecules and cytoskeletal-mediated contraction governs geometrical changes when cells are seeded in these gel-like frameworks (Dean and Morgan, 2008; Krieg et al., 2008; Bao et al., 2011; Youssef et al., 2011). Once seeded, the mold topography and shape of recess mouth affects the stage of cell-settling and tissue self-organization (Svoronos et al., 2014). Keeping this perspective in mind, we used agarose gel molds with 24 recesses that ultimately promoted the formation of rod microtissues of GC and PDLC.

In comparison to other morphologies rods can have several advantages. *In vivo* capillary networks diffuse oxygen and nutrients to thicker tissues approximately 2 mm in width (Griffith et al., 2005) and unlike a spheroid whose final dimensions are governed by diffusion limit, rods can gain any length as long as their width permits capillary diffusion (Dean et al., 2007). However, before this approach can be further investigated basic understanding of the feasibility of control of the geometry over time needs to be gained. The intensity and rate of cell-organization varies with the cell type (Dean et al., 2007; Youssef et al., 2011). For instance, analysis in this study, showed that GC contract to 30% of their length into a stouter kidney-bean-shaped structure, while the PDLC maintained a sleek cylindrical shape, approximately 50% of the original length after 24 h. Time-bound kinetics displayed that a significant contraction in the length of rods was seen 6 h after incubation, which is in synchronization with the kinetics from a similar experimental set up with normal human fibroblasts and H35 cell line revealing a steep contraction curve of rod length contraction in the first 6 h after seeding (Dean et al., 2007). Similar dynamics were observed for spheroids of dental pulp-derived cells where spheroids had formed within 24 h observation period (Janjić et al., 2018b). However, in the setting of Janjić et al. (2018b) unpatterned agarose coating was used without any grooves and therefore the time for microtissue formation is not directly comparable to the approach employed in the current study with 3D Petri dishes^®^.

In addition to the mold pattern and topography, 3D microtissue geometry can be manipulated by introducing elements of tissue constraint (Wang et al., 2013). Contractile forces contribute to an important aspect of the physiological role of various cells such as fibroblasts, smooth muscle cells, cardiomyocytes, epithelial cells, and endothelial cells (Discher et al., 2005; Saez et al., 2005; Ghibaudo et al., 2008; Mitrossilis et al., 2010). These contractile forces restructure the developing tissues by arranging the extracellular matrix. When these tissues are loaded from multiple directions *in vivo*, within their composite peripheral constraints, they acquire mechanical stresses culminating into spatial adaptations in contractility and effecting the morphological alterations in tissues. This principle of tissue constraints has been engaged in the manipulation of 3D microtissues by the introduction of cantilevers (Kalman et al., 2016), pillars, pegs (Youssef et al., 2011), loops, centrifugal, and microelectromechanical forces in the matrix structure (Wang et al., 2013; Ramade et al., 2014; Eyckmans and Chen, 2017). It has been seen that higher magnitude of cell-derived tensile forces have the capability to prevent formation of complex-shaped microtissues. To counteract these forces *in vitro* and allow the formation of stable terminal microtissue structures of the desired shape and size, the amount of extracellular matrix in the tissue can be altered and the stiffness of the constraining structures can be regulated by introducing the above mentioned elements (Wang et al., 2013).

An interesting approach to tailor the contractile forces and 3D tissue morphology is by the application of modulators of cell-signaling pathways acting on the cytoskeletal mediated tension in the 3D tissues (Dean and Morgan, 2008). Previous studies with normal human fibroblasts treated with Rho-kinase inhibitor Y-27632, sorted to the outside of a spheroid when mixed with untreated normal human fibroblasts and formed more stable toroid structure with a slower squeezing around the conical pegs (Dean and Morgan, 2008). Rho-kinase plays a role in cytoskeletal contraction by acto-myosin coupling and phosphorylation (Etienne-Manneville and Hall, 2002). Studies on vascular smooth muscles have shown that activation of the PI3-kinase/Akt pathway in unstimulated smooth muscle have the capability to modify vascular smooth muscle tone, by permitting agonist-induced contraction, through inhibition of the cyclic nucleotide/ HSP20 pathway (Komalavilas et al., 2001). This is in harmony with the findings from our experimental set up, where LY294002, the pharmacological inhibitor of PI3K, was observed to significantly increase the length and decrease the width of both gingival and periodontal rod microtissues, suggesting the role of PI3K pathway in controlling the contraction dynamics of the microtissues *in vitro*.

Thus, we see that deep insights into the contraction dynamics is imperative to establish stable 3D microtissue models for tissue regeneration, biofabrication, and prospective to be used as terminal structures in bioprinting techniques (Datta et al., 2017). Customized 3D microtissues appear to be a promising treatment modality for various fibrotic oral and systemic diseases (Dean and Morgan, 2008), nevertheless, since this is a descriptive study intensive research in cell-signaling mechanisms is required.

## Conclusion

Our findings provide first data on the behavior of microtissue rods derived from gingiva and periodontal ligament cells. Although the geometry of microtissue rods seems to have advantages our data show that the control of the geometry of these microtissues is a challenging issue. Future studies will reveal the optimal architecture of microtissues as building blocks for periodontal tissue engineering.

## Ethics Statement

We used an established protocol for cell isolation. When informed and written consent was given extracted third molars were used to prepare fibroblasts from gingiva and periodontal ligament. The protocol was approved by the Ethics committee of the Medical University of Vienna (631/2007).

## Author Contributions

GO was involved in study design, experimental work, data analysis, data interpretation, manuscript writing. KJ, ASM, OA, and AM were involved in study design, data interpretation, and manuscript writing. BS was involved in experimental work, data analysis, and manuscript writing. HA was involved in study design, data analysis, data interpretation, manuscript writing, manuscript submission, and project supervision.

## Conflict of Interest Statement

The authors declare that the research was conducted in the absence of any commercial or financial relationships that could be construed as a potential conflict of interest.
